# Poster Session II - A224 REAL-WORLD DATA ON THE EFFECTIVENESS OF INTRAVENOUS RISANKIZUMAB RESCUE IN CROHN’S DISEASE

**DOI:** 10.1093/jcag/gwaf042.224

**Published:** 2026-02-13

**Authors:** R E Rosentreter, M O’Brien, M Chan, S Devlin, B Halloran, F Hoentjen, R Ingram, G G Kaplan, K Kroeker, A Lim, K Novak, R Panaccione, C Seow, T Shukla, J St-Pierre, J Siffledeen, K Wong, C Ma, C Lu

**Affiliations:** Medicine, University of Calgary, Calgary, AB, Canada; Medicine, University of Calgary, Calgary, AB, Canada; Medicine, University of Calgary, Calgary, AB, Canada; Medicine, University of Calgary, Calgary, AB, Canada; University of Alberta, Edmonton, AB, Canada; University of Alberta, Edmonton, AB, Canada; Medicine, University of Calgary, Calgary, AB, Canada; Medicine, University of Calgary, Calgary, AB, Canada; University of Alberta, Edmonton, AB, Canada; University of Alberta, Edmonton, AB, Canada; Medicine, University of Calgary, Calgary, AB, Canada; Medicine, University of Calgary, Calgary, AB, Canada; Medicine, University of Calgary, Calgary, AB, Canada; Medicine, University of Calgary, Calgary, AB, Canada; Medicine, University of Calgary, Calgary, AB, Canada; University of Alberta, Edmonton, AB, Canada; University of Alberta, Edmonton, AB, Canada; Medicine, University of Calgary, Calgary, AB, Canada; Medicine, University of Calgary, Calgary, AB, Canada

## Abstract

**Background:**

Risankizumab (RIS) is a monoclonal antibody that targets interleukin 23 for moderate to severe Crohn’s disease (CD). Limited data exist on the efficacy of a single intravenous (IV) rescue dose in CD patients with primary non-response or secondary loss of response.

**Aims:**

In this multicenter retrospective study, we aim to describe the effectiveness of an IV rescue RIS dose in a real-world CD cohort.

**Methods:**

CD patients with a single rescue with RIS (600mg IV) for active disease with C-reactive protein (CRP) ≥8mg/L, fecal calprotectin (FC) >250µg/g, and/or a Harvey Bradshaw Index (HBI) >5 and followed to 3 and 6 months post-rescue when available were included. Primary outcomes are CD patient frequency in clinical remission (HBI score <5), and clinical response (HBI decrease ≥3) at 3 and 6 months. Secondary outcomes are frequency with FC < 250µg/g or CRP <8mg/L.

**Results:**

Thirty-five CD patients (median age 46 years (range 37-66), 9 years CD duration (5-22)) were studied >90 days (range 167-698) post IV rescue. 49% (17) had ileal CD, 17% (6) colonic, and 34% (12) ileocolonic. 34% (12) had inflammatory, 57% (20) stricturing, and 9% (3) fistulizing behaviour. 86% (30) had failed >1 biologic. 20% (7) received corticosteroids at rescue. Median days from initial RIS induction to rescue was 291 (IQR 228-408 days). Of those with baseline HBI >5 and available follow-up HBI scores at 3 months, 33% (3/9) responded, and 11% (1/9) had remission. At 6 months post rescue, 67% (4/6) responded and 50% (3/6) had clinical remission. Of those with baseline FC ≥ 250µg/g (median 592 (437-896) µg/g), 56% (5/9) had a ≥ 50% FC decrease at 3 months and 50% (4/8) at 6 months. Of these, 33% (3/9) and 38% (3/8) had a FC < 250µg/g at 3 and 6 months, respectively. Of those with CRP ≥8mg/L at baseline (median 10.7 (8.5-14.6) mg/L), 80% (4/5) had a CRP <8mg/L (median 5.4 (4.9-6.8) mg/L) and 75% (3/4) (median 4.6 (2.5-7.5) mg/L) at 3 and 6 months, respectively. At a median of 386 days post rescue, 20% (7/35) switched from RIS to a new therapy.

**Conclusions:**

In those with moderate to severe active CD with inadequate response to maintenance RIS, a single 600mg IV rescue may restore disease control. Limitations include small biomarker sample size. Nonetheless, these findings warrant further evaluation of RIS rescue in primary non-response or secondary loss of response.

A224 Table 1: Response Summary

None

**Table:** 

	3 months, N (%)	6 months, N (%)
Clinical Response	3 (33)	4 (67)
Clinical Remission	1 (11)	3 (50)
FC < 250μg/g	3 (33)	3 (38)
FC decrease by ≥ 50%	5 (56)	4 (50)

